# Analyzing COVID-19 Vaccination Side Effects Among the Adult Population in Jeddah, Saudi Arabia

**DOI:** 10.7759/cureus.47136

**Published:** 2023-10-16

**Authors:** Turki Alamri, Fahad Anwer, Nadeem S Butt, Ahmed H Alganmi, Sultan A Alotaibi, Khalid F Alzibali, Hassan A Hawsawi, Marwan Bakarman, Ahmad Azam Malik

**Affiliations:** 1 Department of Family and Community Medicine, Faculty of Medicine in Rabigh, King Abdulaziz University, Jeddah, SAU; 2 Department of Medicine, Faculty of Medicine in Rabigh, King Abdulaziz University, Jeddah, SAU

**Keywords:** covid-19, pandemic, fatigue, side effects, vaccination

## Abstract

The COVID-19 pandemic has brought vaccination to the forefront of global attention. The Pfizer-BioNTech vaccine, an mRNA vaccine that encodes the severe acute respiratory syndrome coronavirus 2 (SARS-CoV-2) glycoprotein spike, has emerged as a significant player in global vaccination efforts. It is generated from lipid nanoparticles and has been subject to various regulatory approvals and authorizations. The United Kingdom became the first country to approve the Pfizer vaccine on December 2, 2020. The World Health Organization (WHO) authorized the emergency use of the Pfizer vaccine on December 31, 2020, facilitating its production and distribution worldwide. In Saudi Arabia, as well as globally, concerns about the safety and effectiveness of vaccines have been raised. Several studies have reported side effects of the Pfizer vaccine, including rare conditions such as myocarditis. In our study, we aimed to systematically investigate the symptoms experienced after vaccination, considering the administration of three doses. We also explored the duration of these symptoms and whether they necessitated hospital visits, primary healthcare interventions, or resolved on their own. Our study employed an online cross-sectional design conducted in Jeddah, Saudi Arabia, utilizing an online self-reported survey. A total of 332 participants who met the predefined criteria were recruited for the study. The rate of COVID-19 infection after 1st and 2nd doses of Pfizer and AstraZeneca vaccines was significantly lower in middle-age subgroups (31-45 years), in comparison to young (18-30 years) and upper middle-age subgroups (46-60 years). For the AstraZeneca vaccine, the infection rate in the middle-aged group was higher after 2nd dose as compared to its 1st dose. Overall, greater infection rates were observed in upper-middle-aged subgroups with all doses of Pfizer and AstraZeneca vaccines. Fatigue and fever were the most common generalized side effects while redness/swelling/pain at the injection site, muscle pain, and joint pain were the most important local side-effects. Fatigue, fever, muscle pain, and joint pain were significantly common after 1st dose of Pfizer and fever was a significant side effect after 2nd dose of Pfizer in comparison to AstraZeneca doses. Understanding the spectrum of side effects associated with the vaccine is crucial for healthcare professionals and individuals receiving the vaccine, as it enables informed decision-making and appropriate management of potential adverse reactions.

## Introduction

Coronavirus disease (COVID-19) is a virus-borne infection caused by the severe acute respiratory syndrome coronavirus 2 (SARS-CoV-2) [1]. This virus has become a worldwide pandemic. In December 2019, in Wuhan City, China, the first COVID-19 case was reported to WHO. Thenceforth, it rapidly spread around the planet [2]. As of December 18, 2022, over 649 million confirmed cases of COVID-19 and over 6.6 million deaths due to COVID-19 infection were reported by WHO [1]. Whereas in Saudi Arabia, where we conducted our research, there were 787,212 cases reported up to June 22, 2022 [3]. Therefore, effective vaccines were extremely necessary to provide strong protection against the virus and its complications, which include serious illness, hospitalization, and death from COVID-19. The world was racing to find a vaccine for the virus, and safety was a huge challenge. As of April 8, 2022, WHO has evaluated several vaccines against COVID-19 that have met the necessary requirements for efficacy and safety (AstraZeneca/Oxford vaccine, Johnson & Johnson, Moderna, Pfizer-BioNTech, Sinopharm, Sinovac, COVAXIN, Covovax, Nuvaxovid, CanSino) [4].

In Saudi Arabia, three vaccines were approved by the Saudi Food and Drug Authority (SFDA). First, the authorities allowed the use of Pfizer on November 24, 2020 [5]. After a few months, on February 18, 2021, SFDA gave approval to register the second vaccine, a viral vector vaccine developed by a British company incorporated with Oxford University, known as AstraZeneca COVID-19 [6]. Later, on April 30, 2021, a third vaccine, the "Moderna COVID-19 vaccine mRNA-1273," was approved for use by WHO [7] and soon after, approved by SFDA for use in the kingdom.

Saudi Arabia promptly took the initiative and provided these three vaccines, primarily Pfizer and AstraZeneca, to the public over three phases. The first phase was aimed at people over the age of 65 and front-line healthcare workers. Phase 2 targeted people over the age of 50 and other healthcare providers. Finally, phase 3 targeted all of Saudi Arabia's population [4].

After the COVID-19 vaccine was approved by the SFDA, it raised many questions and fears about its side effects among the Saudi population and the lack of research and awareness of its side effects. There were 66,700,629 doses of the COVID-19 vaccine given in Saudi Arabia between January 2020 and July 2022 among all the categories of society (old people, children, chronic patients, etc.). A cross-sectional study was carried out at King Khalid University, Abha, Aseer region of the Kingdom of Saudi Arabia, from March to May of 2021, to study the adverse effects of the Pfizer and AstraZeneca vaccines. The participants (226 out of 330) experienced systemic adverse effects from these two types of vaccines such as fever, fatigue, headache, and muscle and joint pain, but very few reported injection site redness or other symptoms. Fever was the most prevalent symptom [8]. There was also another study assessing the side effects of the different COVID-19 vaccines among the Saudi population. The majority of participants (87.5%) experienced at least one of the reported side effects, with most of the common symptoms similar to the previous study, while (12.5%) had no side effects [9].

The main challenge of previous studies is the time of data collection period, the Saudi government only allowed people who were over 60 years old, healthcare workers, and those with a few selected medical conditions, to take the second dose of the vaccine [9]. Most of the symptoms reported above occurred in the early phase after vaccination. delayed side effects of the vaccine which appear after a period from taking the dose were not included in these previous studies. There is no specific duration of the onset of symptoms after vaccine administration [10]. Also, the third booster dose wasn’t included in previous studies. 

This study aimed to assess the side effects of COVID-19 vaccines (Pfizer, AstraZeneca, and Moderna) among adults in the Jeddah population who received three doses of the COVID-19 vaccine. The study will add to the existing knowledge related to vaccine safety and will be useful for healthcare providers to increase their awareness regarding immediate and delayed adverse effects of the COVID-19 vaccines.

## Materials and methods

This study was a cross-sectional survey conducted online in Jeddah, Saudi Arabia, beginning in September 2022 and continuing for 3 months. Participants were recruited through social media platforms including WhatsApp, Twitter, and Instagram, using survey links. Those who had chronic disease, under 18 years of age, or over 60 years of age were excluded from the study before participating in the survey. If a participant did not complete the questionnaire form, they were withdrawn from the study. The questionnaire assessed the personal and demographic data, health condition, COVID-19 vaccine information, and side effects history.

Data were analyzed using the Statistical Package for Social Sciences (SPSS) program version 26 (IBM Corp., Armonk, NY). Qualitative data were expressed as frequencies and percentages, and Quantitative data were expressed in terms of their mean and standard deviation (Mean ± SD). The Chi-square and Fisher’s Exact tests (χ2) were used to assess associations between the outcome variable (side-effects of vaccine) and various study variables. A p-value of less than 0.05 was considered statistically significant.

The sample size of 332 was estimated using the Raosoft calculator with a confidence level of 95% and a 5% margin of error. Ethical approval was provided by the Research Ethics Committee of the College of Medicine, King Abdulaziz University (Reference No. 457-22). 

## Results

This study included 332 participants who completed the survey. The percentage of females was 41.3% and males 58.7%. While 27.4% were between the ages of 31 and 45, 64.2% were between 18 and 30; 96.4% were Saudi, and 67.2% were high school graduates or less. 

The descriptive social and demographic statistics (Table 1) display the percentages of the participants' distribution by age groups, gender, nationality, level of education, and the location of their residence in Jeddah.

**Table 1:** Descriptive social and demographic statistics

		n	%
Age category	18-30	213	64.2%
	31-45	91	27.4%
	46-60	28	8.4%
Chronic disease	No	332	100.0%
	Yes	0	0.0%
Gender	F	137	41.3%
	M	195	58.7%
Your nationality	Saudi	320	96.4%
	Non-Saudi	12	3.6%
Educational level	High school or less	54	16.3%
	Graduate	223	67.2%
	Postgraduate studies	55	16.6%
Residence in Jeddah	North Jeddah	139	41.9%
	East Jeddah	55	16.6%
	South Jeddah	57	17.2%
	West Jeddah	20	6.0%
	Center Jeddah	61	18.4%

**Figure 1:**
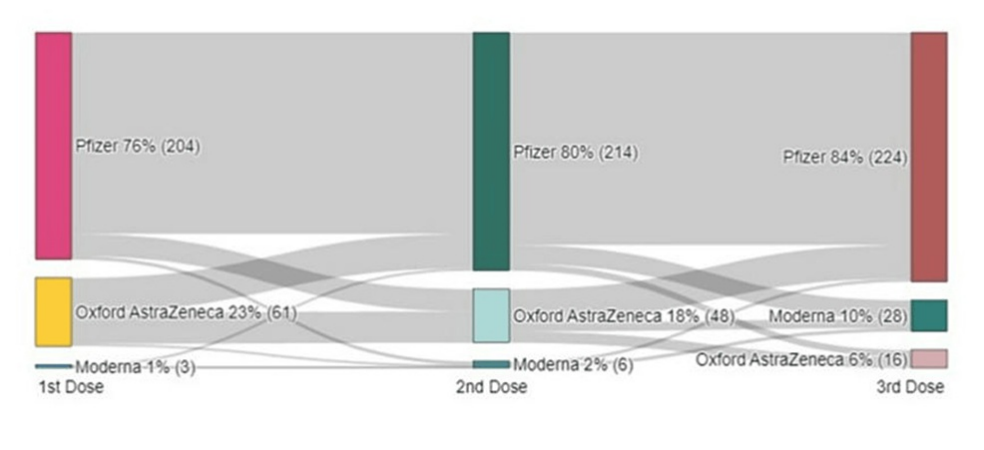
Sankey diagram for the trends of received COVID-19 vaccines

Figure 1 describes the details of the uptake of various vaccines over different doses. Pfizer vaccine was received by the majority (1st dose n=204, 76%) of the study sample and an increasing trend in choice of Pfizer was observed at 80% and 84% in 2nd and 3rd doses respectively. However, a slight shift from Pfizer to Oxford AstraZeneca was observed from 1st dose to 2nd dose but mostly shifted to Pfizer or Moderna for the 3rd dose of vaccine. 

**Figure 2:**
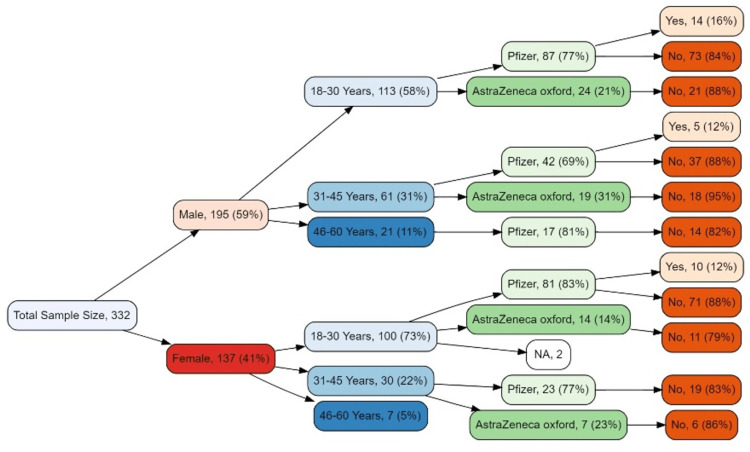
Tree diagram for infection rate after 1st dose by vaccine type, age, and gender Frequencies <5 at each node were not included in the tree diagram

Figure 2 describes the details of the type of COVID-19 vaccine received and the infection rate after the 1st dose. There were 87 young (18-30 years) male respondents who received Pfizer, approximately (16%, n=14) of this subgroup got COVID-19 positive after 1st dose. A relatively lower infection rate (12%) was observed in this subgroup of those who received Oxford AstraZeneca. In the young (18-30 years) female subgroup infection rate after 1st dose of Pfizer and AstraZeneca vaccines was observed to be 12% and 21% respectively. In the middle-aged (31-45 years) male subgroup, only 5% got infected after receiving Pfizer and Oxford AstraZeneca. However, in the middle-aged (31-45 years) female subgroup, nearly similar infection rates were observed at 17% and 14% with Pfizer and Oxford AstraZeneca respectively. Considerably greater infection rates were observed in the upper middle-aged (40-60 years) male subgroup of the study sample with both Pfizer and Oxford AstraZeneca at 18% and 25% respectively.

**Figure 3:**
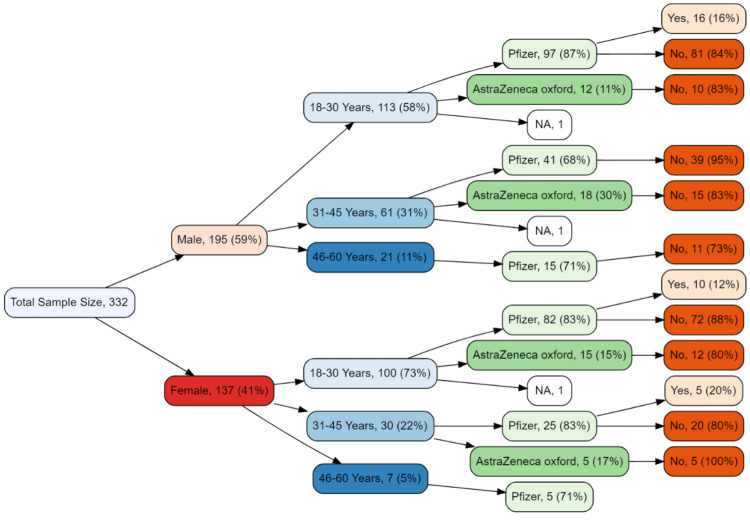
Tree diagram for infection rate after 2nd dose by vaccine type, age, and gender Frequencies <5 were not included in the tree diagram

Figure 3 describes the details of the type of COVID-19 vaccine received and the infection rate after the 2nd dose. There were 97 young (18-30 years) male respondents who received Pfizer; approximately 16% of this subgroup got COVID-19 positive after 2nd dose. A similar infection rate (16.66%) was observed in this subgroup of those who received Oxford AstraZeneca. In the young (18-30) female subgroup infection rate after 2nd dose of Pfizer and AstraZeneca vaccines was observed to be 12% and 20% respectively. In the middle-aged (31-45 years) male subgroup, only 5% got infected after receiving Pfizer while a relatively higher infection rate was observed (17%) among those who received Oxford AstraZeneca. However, in the middle-aged female subgroup similar infection rates were observed with both Pfizer and Oxford AstraZeneca. Considerably greater infection rates were observed in the upper middle-aged (40-60 years) subgroup of the study sample with both Pfizer and Oxford AstraZeneca at 27% and 25% respectively.

**Figure 4:**
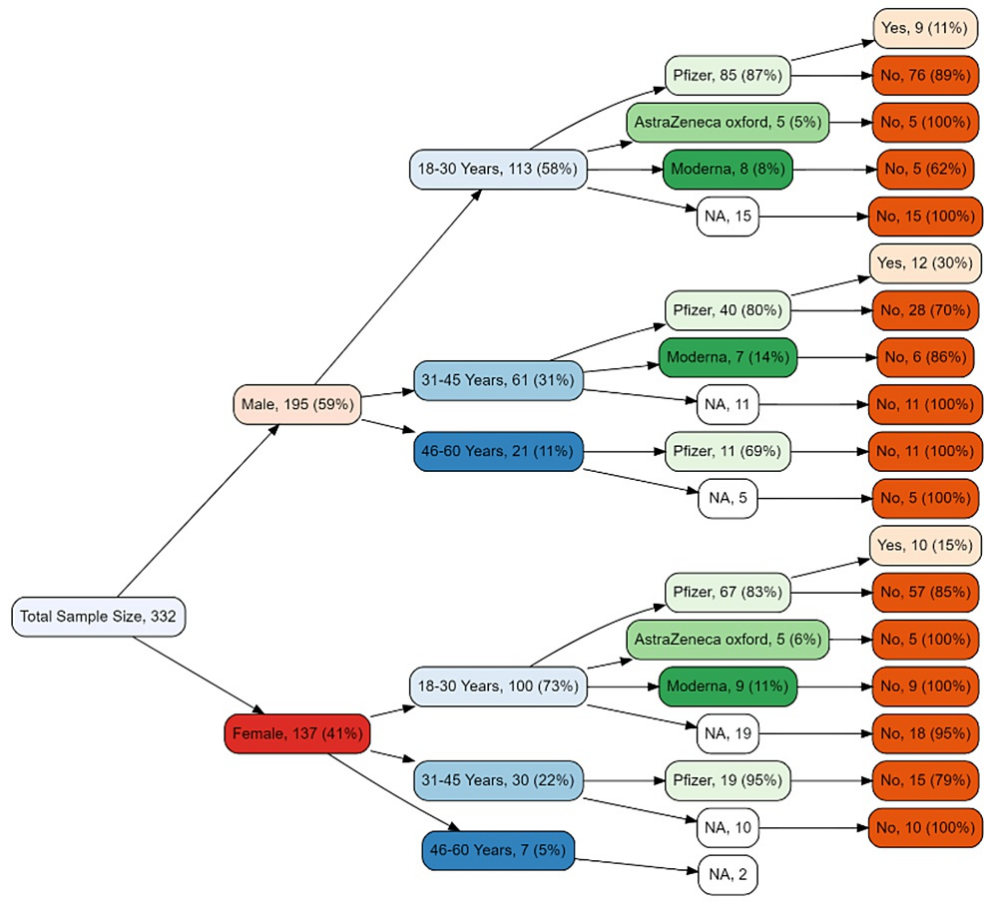
Tree diagram for infection rate after 3rd dose by vaccine type, age, and gender Frequencies <5 were not included in the tree diagram

Figure 4 describes the details of the type of COVID-19 vaccine received and the infection rate after the 3rd dose. There were 85 young (18-30 years) male respondents who received Pfizer, 11% of this subgroup got COVID-19 positive after the 3rd dose, which is relatively lower as compared to the 1st and 2nd doses of Pfizer. There were few respondents in this group that received Oxford AstraZeneca (n=5) and none of them got COVID-19 positive after the 3rd dose. In the young (18-30) female subgroup (n=67), the infection rate after 3rd dose of Pfizer and AstraZeneca vaccines was observed to be 15% and 0% respectively. In the middle-aged (31-45 years) male subgroup (n=40), after 3rd dose of Pfizer very high infection rate was observed (n=12, 30%) while in same aged female subgroup, it was observed to be 21%.

Our study results (Table 2) illustrate the reported side effects, some recipients reported redness/swelling/pain at the site of injection, 113 out of a total of 254 after the 1st dose, 81 out of 265 after the 2nd dose, and 60 out of 225 after the 3rd dose. Also, for musculoskeletal symptoms, muscle pain was detected as a side effect of the vaccine reported by 89 of 254 after 1st dose, 70 out of 265 after 2nd dose, and 47 out of 225 after 3rd dose, as for joint pain 76 cases reported out of 254 after 1st dose, 60 of 265 after 2nd dose and 49 of 225 after the 3rd dose. Comparing those same adverse effects caused by Pfizer to AstraZeneca vaccine the results were as follows, for redness/swelling/pain at the site of injection, 38 out of a total of 71 after the 1st dose, 14 of 56 after the 2nd dose and 2 of 17 after the 3rd dose. Muscle pain was detected as a side effect for the AstraZeneca vaccine, reported by 41 of 71 after 1st dose, 19 of 56 after 2nd dose, and 2 of 17 after 3rd dose. As for joint pain, 41 cases were reported out of 71 after 1st dose, 19 of 56 after 2nd dose, and 1 of 17 after the 3rd dose.

**Table 2:** Descriptive statistics and comparison between Pfizer and AstraZeneca on vaccination side effects

	Pfizer			Oxford AstraZeneca			Comparison of Side-Effects Pfizer vs Oxford AstraZeneca		
	1^st^	2^nd^	3^rd^	1^st^	2^nd^	3^rd^			
No. vaccinated	254	265	225	71	56	17			
Covid positive after n(%)	36(12.2)	37(14.0)	34(15.1)	9(12.7)	10(17.9)	3(17.6)	1st Dose	2nd Dose	34d Dose
Side-effects	n(%)			n(%)			P-Value	P-Value	P-Value
Fatigue	117(46.1)	86(32.5)	67(29.8)	46(64.8)	22(39.3)	3(17.6)	0.005	0.327	0.289
Redness/swelling/pain at site of injection	113(44.5)	81(30.6)	60(26.7)	38(53.5)	14(25)	2(11.8)	0.177	0.829	0.174
Fever	95(37.4)	13(4.9)	45(20)	42(59.2)	19(33.9)	4(23.5)	0.001	0.000	0.726
Muscle pain	89(35)	70(26.4)	47(20.9)	41(57.7)	19(33.9)	2(11.8)	0.001	0.254	0.368
Joint pain	76(29.9)	60(22.6)	49(21.8)	41(57.7)	19(33.9)	1(5.9)	0.000	0.075	0.119
Headache	80(31.5)	57(21.5)	42(18.7)	34(47.9)	21(37.5)	3(17.6)	0.105	0.111	0.920
Hair loss	43(16.9)	32(12.1)	31(13.8)	10(14.1)	11(19.6)	1(5.9)	0.569	0.131	0.352
Sweating	32(12.6)	25(9.4)	21(9.3)	16(22.5)	7(12.5)	1(5.9)	0.366	0.484	0.631
Sleep disturbance	31(12.2)	25(9.4)	16(7.1)	14(19.7)	11(19.6)	2(11.8)	0.105	0.028	0.478
Loss of appetite	29(11.4)	19(7.2)	16(7.1)	12(16.9)	8(14.3)	2(11.8)	0.219	0.082	0.478
Palpitations	27(10.6)	21(7.9)	15(6.7)	10(14.1)	6(10.7)	1(5.9)	0.418	0.497	0.897
Irregular menstrual cycle	25(9.8)	25(9.4)	20(8.9)	4(5.6)	4(7.1)	1(5.9)	0.271	0.589	0.674
Nausea	31(12.2)	16(6)	12(5.3)	11(15.5)	6(10.7)	1(5.9)	0.465	0.208	0.920
Shortness of breath	23(9.1)	11(4.2)	6(2.7)	6(8.5)	4(7.1)	0(0)	0.873	0.337	0.497
Chest pain	22(8.7)	10(3.8)	7(3.1)	4(5.6)	4(7.1)	0(0)	0.407	0.263	0.459
Others	9(3.5)	13(4.9)	10(4.4)	2(2.8)	2(3.6)	0(0)	0.764	0.667	0.373
Amenorrhea	11(4.3)	12(4.5)	9(4)	0(0)	2(3.6)	0(0)	0.075	0.749	0.467
Weight gain	11(4.3)	6(2.3)	13(5.8)	3(4.2)	1(1.8)	0(0)	0.968	0.826	0.308
Weight loss	7(2.8)	5(1.9)	7(3.1)	1(1.4)	3(5.4)	0(0)	0.516	0.131	0.459
Increase in appetite	9(3.5)	10(3.8)	6(2.7)	3(4.2)	0(0)	0(0)	0.787	0.139	0.497
Sexual problems	7(2.8)	3(1.1)	2(0.9)	2(2.8)	1(1.8)	0(0)	0.976	0.689	0.697

Regarding cardiological symptoms, around 27 patients suffered palpitation of 254 patients who received the 1st dose of Pfizer vaccine, where 21 of 265 after the 2nd dose of Pfizer reported palpitation and 15 of 225 reported palpitation after the 3rd dose. Recipients who suffered chest pain after being vaccinated with the Pfizer vaccine were 22 of 254 after the 1st dose, 10 of 265 after the 2nd dose, and 7 of 225 after the 3rd dose. Results of the AstraZeneca vaccine vary greatly, for recipients who suffered palpitation, 10 of 71 after the 1st dose, 6 of 56 after the 2nd dose, and 1 of 17 after the 3rd dose. Patients who reported chest pain after the AstraZeneca vaccine were 4 of 71 after the 1st dose, 4 of 56 after the 2nd dose, and 0 of 17 after the 3rd dose.

Going to menstrual problems, our study reported irregular menstrual cycles following the Pfizer vaccine, where 25 patients out of 254 reported after 1st dose, 25 out of 265 after 2nd dose, and 20 out of 225 after 3rd dose. Amenorrhea was reported in 11 of 254 after 1st dose, 12 of 265 after 2nd dose, and 9 of 225 after 3rd dose. Following AstraZeneca, irregular menstrual cycle was reported, where 4 patients reported 71 after 1st dose, 4 of 56 after 2nd dose, and 1 of 17 after 3rd dose. Amenorrhea was negligible with AstraZeneca.

**Figure 5:**
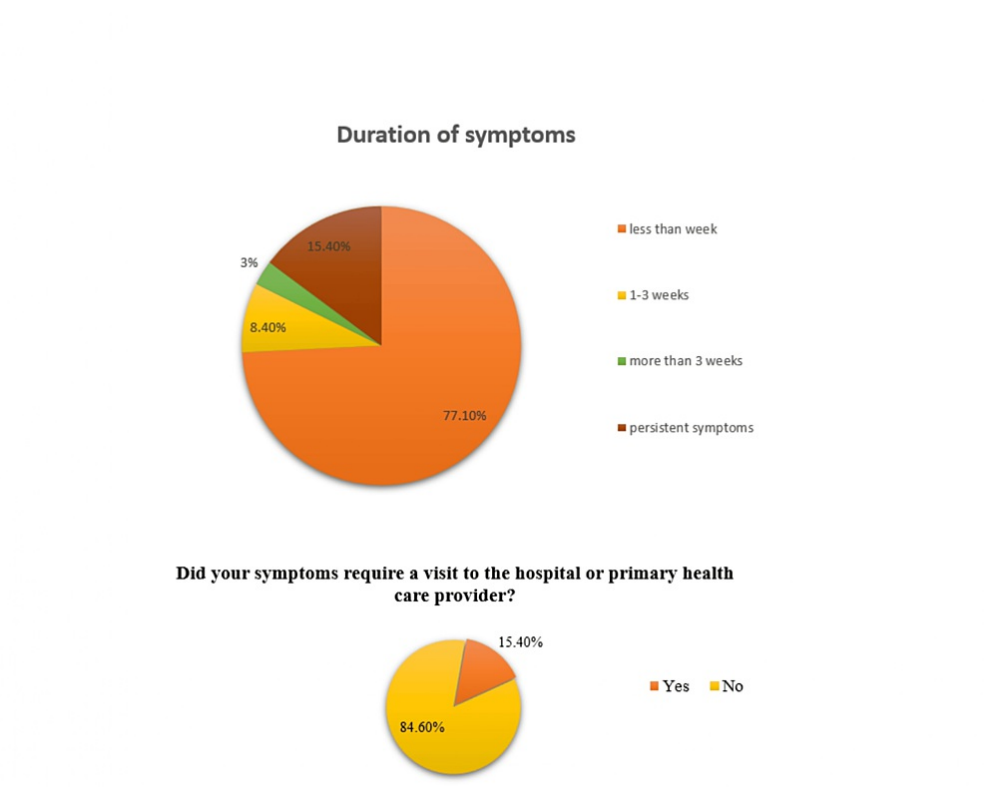
Period of experience of symptoms and whatever needed hospital or primary health care or resolved spontaneously

As illustrated in Figure 5, 77.1% of participants reported suffering from side effects less than one week of vaccination, 8.4% in one to three weeks, 3% in more than three weeks, and 11.4% reported persistent symptoms. 84.6% reported that symptoms do not require a visit to the hospital or primary health care.

## Discussion

Researchers from all over the world put efforts to identify a vaccine candidate that could be used to combat the recent coronavirus outbreak. However, it is important to remember that making a vaccine is not simple or quick. A vaccination must go through three stages of clinical trials to demonstrate its safety and efficacy before being made available to the general public [11]. Because of the inflammatory reaction to vaccination, vaccine reactogenicity refers to a variety of local and systemic symptoms. The reactogenicity is influenced by several variables, including host characteristics (age, gender, etc.), vaccination type, composition, delivery route, and many more. As a result, it is expected that most people will experience a vaccine reaction after receiving a COVID-19 vaccination [11].

Liu et al. demonstrated that there were 4 cases of myocarditis reported at the Duke University Medical Center in Durham out of 561,197 people who received the Pfizer vaccine and Moderna vaccine; all 4 patients were later admitted to hospitals after experiencing cardiological symptoms [12]. According to studies, the most common cardiac side effect of the mRNA COVID-19 vaccination (both Pizer and Moderna), particularly in young adult men, was myocarditis/myopericarditis. Additionally, people who received the Pfizer vaccine, particularly the second dosage, were more prone to myocarditis/myopericarditis than people who received the Moderna vaccine [13,14]. Due to spontaneous reports of thromboembolic incidents among vaccine recipients, numerous European nations ceased using the AstraZeneca vaccine in March 2021 [15]. According to two different studies conducted on people who had received the AstraZeneca vaccine, the standardized morbidity ratio for thromboembolic events was greater in this study cohort than in the general population [16,17]. Comparing those results to our study results, we find out participants who received Pfizer were more prone to chest pain while AstraZeneca vaccine receivers complained more from palpitations. The P-values were insignificant according to our study results.

According to our study results, fatigue was the most reported post-vaccination side effect followed by redness or swelling at the site of injection, fever, muscle pain, joint pain, headache, hair loss, sweating, sleep disturbance, loss of appetite, palpitation. These side effects were reported by both the Pfizer vaccine and the Oxford AstraZeneca vaccine. According to a study done in the United Arab Emirates, the COVID-19 vaccine caused some negative reactions in almost 65% of the study's participants [18]. Pain at the injection site, fever, exhaustion, and headache were the most frequent side effects reported by recipients of the inactivated and mRNA vaccines, respectively. For recipients of the Pfizer vaccine, these side effects were followed by fatigue and headache. The most uncommon side effect among Sinopharm vaccine recipients was enlarged, swollen lymph nodes, followed by a change in or loss of taste, while among Pfizer vaccine recipients, one participant experienced temporary one-sided facial weakness before experiencing enlarged, swollen lymph nodes [18]. The adverse effects of the Moderna vaccination were studied by Kadali et al. [10]. According to a poll of 432 healthcare workers who had received the vaccination, adverse effects included muscle spasms, poor sleep, brain fog, flushing, heat/cold intolerance, and palpitations. Local edema and myalgia were also very common [10]. Another study conducted among 395 AstraZeneca-vaccinated individuals in Ethiopia found that 39.24% of people experienced pain at the injection site as a local symptom, while 43.4% of people reported muscle pain as a systemic symptom [19].

In phase 3 clinical trials of the Pfizer vaccine, the most frequent side effects following the initial dosage were pain at the injection site (71-83%), fatigue (34-47%], and headache (25-42%) [20]. However, a community review in the UK indicated that systemic side effects, such as fatigue and headache, impacted fewer than one in four patients and were less prevalent in the population than predicted from clinical studies [15]. After the initial dose, fewer than 25% of users reported fatigue and headaches, while less than 30% of users reported pain at the injection site. In a phase 3 trial for that vaccine, 51-59% of participants reported feeling fatigued after receiving the second dose of the Pfizer vaccine [20]. However, in UK research, less than 15% of individuals reported feeling fatigued after receiving the second dose [21]. In research from Saudi Arabia on those who received the Pfizer vaccine, similar uncommon side events were also documented [22]. However, the percentage found in this investigation was higher than that reported in the trials [23,24]. However, additional studies based on actual data have revealed a comparable proportion of unfavorable outcomes in both inactivated and mRNA vaccination recipients [25,26].

The vaccine from AstraZeneca is created from replication-deficient chimpanzee adenovirus ChAdOx1. It contains the SARS-CoV-2 structural surface spike protein gene. Four randomized clinical trials conducted on AstraZeneca in the UK, South Africa, and Brazil have produced results on its efficacy and safety. All four studies found the vaccination to be generally safe, and the frequency of major adverse events was similar in each research group. One hundred and sixty-eight severe adverse events were observed overall among 79 AstraZeneca users and 89 saline control recipients [27].

Parallel to this, the AstraZeneca vaccine's side-effect rates were lower than anticipated [28]. The AstraZeneca vaccine phase 2-3 trial [19] reported that 88% of participants aged 18 to 55 who got the first injection experienced systemic side effects, whereas we discovered a lower rate of 337% after the first dose in the entire population and 469%. Systemic side effects were more common in people who received the AstraZeneca vaccine than in people who received the Pfizer vaccine, but in a different study, 89% of respondents who logged at least one systemic effect after receiving the AstraZeneca vaccine did not report any after 3 days, and 983% did not report any after 1 week [25].

Continuingly, our study results illustrate that side effects after both vaccines were higher after receiving the 1st dose compared to the 2nd and 3rd dose which had the least number of side effects. Although for menstrual irregularity, it reported continuously after each dose with nearly similar numbers which is a new finding for this study. However, according to two retrospective studies conducted in Saudi Arabia and the UK, respectively, menstrual irregularities were observed to have a low incidence and a low likelihood of being related to COVID-19 vaccination. In these trials, it was anticipated that menstrual irregularities might be explained by underlying platelet/clotting issues or by the fact that women receiving the vaccine doses already had delayed menstrual cycles [29,30].

Pharmacovigilance post-marketing is essential to monitoring vaccines as it is a crucial component of assessing their effectiveness and safety, especially in high-risk populations. New preventative strategies such as antiviral treatments, medications that may slow the progression of the disease, monoclonal antibodies, hyperimmune globulin, and convalescent titers are anticipated to be developed. If these methods are found to be successful, they may be applied to high-risk individuals such as older people and healthcare personnel. It's crucial to continue using preventative measures like often washing hands with soap and water or gel alcohol and covering your mouth when coughing or sneezing.

We find in the availability of vaccine types, the Pfizer vaccine was the first one made available in the Kingdom of Saudi Arabia, and it was also the most widely distributed, so many people were able to receive it. However, for the second and third doses, the vaccine types were expanded and made available in the Kingdom of Saudi Arabia. Additionally, Pfizer built a reputation for vaccination safety after receiving the first dosage which encouraged them to continue receiving Pfizer's second and third doses. Additional findings indicate significantly higher infection rates in the upper middle-aged (40-60 years) male subgroup of the study sample for both the Pfizer and Oxford AstraZeneca vaccines. In the first dose, infection rates were 18% for Pfizer and 25% for Oxford AstraZeneca, while in the second dose, infection rates rose to 27% for Pfizer and remained at 25% for Oxford AstraZeneca within the same subgroup. Further investigation is required to understand the underlying factors contributing to these differences in vaccine efficacy among upper middle-aged males and to inform targeted vaccination strategies. Following the third dosage of Pfizer, AstraZeneca, or Moderna vaccines, infection rates were found to be 15% in those who receive Pfizer and 0% for both AstraZeneca and Moderna. The lack of participants who received the AstraZeneca and Moderna could be the reason for the infection rate percentage.

Limitation

The results of this study add to the national data by supplying actual, personally reported information on the adverse effects of the COVID-19 vaccination. However, a few restrictions need to be considered while interpreting our results. First, we discuss the adverse effects mentioned in the questionnaire that was given out. However, given how quickly the vaccine was created and how its efficacy was shown, there might be other unrecognized negative consequences limited by the sample size and might show up in bigger geographic areas. Also, Moderna vaccination was not approved as rapidly as Pfizer and AstraZeneca in Saudi Arabia, so the number of doses that were given was not enough. Additionally, some participants reported the symptoms a year after taking the vaccine, recall bias may exist. And lastly, the survey's online dissemination method could lead to selection bias.

## Conclusions

In summary, the study found that COVID-19 vaccines, Pfizer and AstraZeneca, had different side effects, with fatigue being the most common followed by pain/redness at the injection site and fever. These symptoms were significant for Pfizer's 1st dose as compared to AstraZeneca's 1st dose. Reinfection was more common in the 46 to 60 years age group and least common in the 31-45 years age group after each of the three vaccine doses Most of the reported symptoms were mild and did not require hospital intervention. The study highlights the importance of monitoring side effects and expanding research on vaccine safety and effectiveness. However, monitoring over a longer period may shed light on potential future negative reactions and rule out those that are incorrectly attributed to vaccinations. In order to develop the proper policies on safe vaccination use it is critical to identify the underlying immunologic and nonimmunologic processes of adverse events.

## References

[1] (2022). Coronavirus disease (COVID-19). https://www.who.int/health-topics/coronavirus.

[2] Yüce M, Filiztekin E, Özkaya KG (2021). COVID-19 diagnosis-a review of current methods. Biosens Bioelectron.

[3] (2022). Ministry statistics. https://www.moh.gov.sa/en/Pages/default.aspx.

[4] (2022). COVID-19 advice for the public: getting vaccinated. updated.

[5] (2022). SFDA approves the registration of the coronavirus vaccine "Pfizer-BioNTech". https://www.sfda.gov.sa/ar/news/73864.

[6] (2022). Saudi Food & Drug Authority allows the import and use of AstraZeneca Covid19 vaccine. Kingdom.

[7] (2022). WHO lists Moderna vaccine for emergency use. https://www.who.int/news/item/30-04-2021-who-lists-moderna-vaccine-for-emergency-use.

[8] Adam M, Gameraddin M, Alelyani M (2021). Evaluation of post-vaccination symptoms of two common COVID-19 vaccines used in abha, aseer region, kingdom of Saudi Arabia. Patient Prefer Adherence.

[9] Almughais ES, Alharbi AH, Aldarwish HA, Alshammari AF, Alsuhaymi RS, Almuaili JA, Alanizy AM (2022). Side-effects of COVID-19 vaccines among the Saudi population: a cross-sectional study. Saudi Med J.

[10] Kadali RA, Janagama R, Peruru S, Malayala SV (2021). Side effects of BNT162b2 mRNA COVID-19 vaccine: a randomized, cross-sectional study with detailed self-reported symptoms from healthcare workers. Int J Infect Dis.

[11] Hervé C, Laupèze B, Del Giudice G, Didierlaurent AM, Tavares Da Silva F (2019). The how's and what's of vaccine reactogenicity. NPJ Vaccines.

[12] Liu R, Pan J, Zhang C, Sun X (2022). Cardiovascular complications of COVID-19 vaccines. Front Cardiovasc Med.

[13] Kim HW, Jenista ER, Wendell DC (2021). Patients with acute myocarditis following mRNA COVID-19 vaccination. JAMA Cardiol.

[14] Fazlollahi A, Zahmatyar M, Noori M (2022). Cardiac complications following mRNA COVID-19 vaccines: a systematic review of case reports and case series. Rev Med Virol.

[15] Wise J (2021). Covid-19: European countries suspend use of Oxford-AstraZeneca vaccine after reports of blood clots. BMJ.

[16] Mahase E (2021). AstraZeneca vaccine: blood clots are "extremely rare" and benefits outweigh risks, regulators conclude. BMJ.

[17] Pottegård A, Lund LC, Karlstad Ø (2021). Arterial events, venous thromboembolism, thrombocytopenia, and bleeding after vaccination with Oxford-AstraZeneca ChAdOx1-S in Denmark and Norway: population based cohort study. BMJ.

[18] Ganesan S, Al Ketbi LM, Al Kaabi N (2022). Vaccine side effects following COVID-19 vaccination among the residents of the UAE-an observational study. Front Public Health.

[19] Tequare MH, Abraha HE, Adhana MT (2021). Adverse events of Oxford/AstraZeneca's COVID-19 vaccine among health care workers of Ayder Comprehensive Specialized Hospital, Tigray, Ethiopia. IJID Reg.

[20] Polack FP, Thomas SJ, Kitchin N (2020). Safety and efficacy of the BNT162b2 mRNA Covid-19 vaccine. N Engl J Med.

[21] Menni C, Klaser K, May A (2021). Vaccine side-effects and SARS-CoV-2 infection after vaccination in users of the COVID Symptom Study app in the UK: a prospective observational study. Lancet Infect Dis.

[22] El-Shitany NA, Harakeh S, Badr-Eldin SM (2021). Minor to moderate side effects of Pfizer-BioNTech COVID-19 vaccine among Saudi residents: a retrospective cross-sectional study. Int J Gen Med.

[23] Xia S, Duan K, Zhang Y (2020). Effect of an inactivated vaccine against SARS-CoV-2 on safety and immunogenicity outcomes: interim analysis of 2 randomized clinical trials. JAMA.

[24] Mulligan MJ, Lyke KE, Kitchin N (2020). Phase I/II study of COVID-19 RNA vaccine BNT162b1 in adults. Nature.

[25] Saeed BQ, Al-Shahrabi R, Alhaj SS, Alkokhardi ZM, Adrees AO (2021). Side effects and perceptions following Sinopharm COVID-19 vaccination. Int J Infect Dis.

[26] Zhang MX, Zhang TT, Shi GF, Cheng FM, Zheng YM, Tung TH, Chen HX (2021). Safety of an inactivated SARS-CoV-2 vaccine among healthcare workers in China. Expert Rev Vaccines.

[27] Voysey M, Clemens SA, Madhi SA (2021). Safety and efficacy of the ChAdOx1 nCoV-19 vaccine (AZD1222) against SARS-CoV-2: an interim analysis of four randomised controlled trials in Brazil, South Africa, and the UK. Lancet.

[28] Oliver SE, Gargano JW, Marin M (2020). The Advisory Committee on Immunization Practices’ interim recommendation for use of Pfizer-BioNTech COVID-19 vaccine - United States, December 2020. MMWR Morb Mortal Wkly Rep.

[29] Alghamdi AN, Alotaibi MI, Alqahtani AS, Al Aboud D, Abdel-Moneim AS (2021). BNT162b2 and ChAdOx1 SARS-CoV-2 post-vaccination side-effects among Saudi vaccinees. Front Med (Lausanne).

[30] Male V (2021). Effect of COVID-19 vaccination on the timing and flow of menstrual periods in two cohorts. medRxiv.

